# Fast, accurate, and racially unbiased pan-cancer tumor-only variant calling with tabular machine learning

**DOI:** 10.1038/s41698-022-00340-1

**Published:** 2023-01-07

**Authors:** R. Tyler McLaughlin, Maansi Asthana, Marc Di Meo, Michele Ceccarelli, Howard J. Jacob, David L. Masica

**Affiliations:** 1 Genomics Research Center, AbbVie, Redwood City, CA USA; 2 Agricultural and Biological Engineering at Purdue University, West Lafayette, IN USA; 3 Johns Hopkins University, Baltimore, MD USA; 4 Department of Electrical Engineering and Information Technology, University of Naples “Federico II”, Naples, Italy; 5 Biogem, Instituto di Biologia e Genetica Molecolare, Ariano Irpino, Italy

**Keywords:** Cancer genomics, Computational biology and bioinformatics

## Abstract

Accurately identifying somatic mutations is essential for precision oncology and crucial for calculating tumor-mutational burden (TMB), an important predictor of response to immunotherapy. For tumor-only variant calling (i.e., when the cancer biopsy but not the patient’s normal tissue sample is sequenced), accurately distinguishing somatic mutations from germline variants is a challenging problem that, when unaddressed, results in unreliable, biased, and inflated TMB estimates. Here, we apply machine learning to the task of somatic vs germline classification in tumor-only solid tumor samples using TabNet, XGBoost, and LightGBM, three machine-learning models for tabular data. We constructed a training set for supervised classification using features derived exclusively from tumor-only variant calling and drawing somatic and germline truth labels from an independent pipeline using the patient-matched normal samples. All three trained models achieved state-of-the-art performance on two holdout test datasets: a TCGA dataset including sarcoma, breast adenocarcinoma, and endometrial carcinoma samples (AUC > 94%), and a metastatic melanoma dataset (AUC > 85%). Concordance between matched-normal and tumor-only TMB improves from *R*^*2*^ = 0.006 to 0.71–0.76 with the addition of a machine-learning classifier, with LightGBM performing best. Notably, these machine-learning models generalize across cancer subtypes and capture kits with a call rate of 100%. We reproduce the recent finding that tumor-only TMB estimates for Black patients are extremely inflated relative to that of white patients due to the racial biases of germline databases. We show that our approach with XGBoost and LightGBM eliminates this significant racial bias in tumor-only variant calling.

## Introduction

An important application of somatic variant calling is patient selection in cancer immunotherapy clinical trials because somatic mutation count can predict response to immune checkpoint inhibitors (ICI)^1–3^. Tumor mutation burden (TMB)—defined as the number of coding nonsynonymous somatic mutations per megabase of DNA, and often measured through whole-exome sequencing (WES)—is a strong predictor of therapeutic response and survival in solid tumors. Encouraged by recent results from the successful phase 2 KEYNOTE-158 trial^4^, the FDA has approved TMB as a marker across all tumor subtypes for the anti-PD1 ICI pembrolizumab, where higher TMB is associated with an increased likelihood of benefit. This approval broadens the importance of reliably estimating patient-level TMB using WES data.

In addition to TMB, somatic and germline variants is used to understand the molecular basis of cancer. Somatic mutation underlies cancer formation and progression, often through gain-of-function mutations in oncogenes and loss-of-function mutations in tumor suppressors^5^. It is becoming increasingly crucial to characterize and identify somatic mutations to predict whether a cancer patient will be resistant or responsive to existing targeted therapies. Germline variation in genes such as BRCA and TP53 can also be heritable cancer drivers, so understanding the germline context of cancer can complement the characterization of acquired somatic mutations.

Matched-normal samples are not always available in the clinic, leading to entirely tumor-only cohorts and mixed cohorts of tumor-only and matched-normal samples. Causes for missing a matched-normal sample include failed quality control in the normal samples and a lack of consent to procure blood samples for germline variant analysis. Furthermore, the acquisition of a patient’s matched normal must be included in the design of the oncology clinical trial, which is not a routine practice.

The absence of a patient-matched normal complicates somatic variant calling in precision oncology. The sheer number of rare germline variants per sample and their broad distribution of variant allele fractions (VAFs) (Supplementary Fig. 1) makes it challenging to retrieve the relatively small number of genuine somatic mutations. One study reported the absence of a matched-normal sample leads to a 67% false positive rate; thus, most putative somatic mutations in tumor-only variant calling are instead rare germline variants^6^. The resulting tumor-only TMB estimate is artificially inflated relative to “true” TMB derived via germline variant subtraction using a matched-normal. One recent study reported a fold inflation of 2.2–16.9 for tumor-only-calculated TMB, depending on the chosen germline database-filtering strategy^7^.

Several computational methods have been developed to improve tumor-only variant calling, either by sophisticated filtering approaches^8^, or via explicit statistical inference of the somatic alteration state of the cancer genome (by algorithms like ABSOLUTE^9^ and CLONET^10^. The latter category includes PureCN^11,12^ and SGZ^13^, two recently developed Bayesian methods that infer the altered genomic state of the tumor to estimate somatic and germline probabilities in samples without a matched normal. These methods first estimate global properties of the cancer genome (purity and ploidy) as well as local DNA copy number. They integrate this information with the observed variant allele frequencies (VAFs) to calculate the posterior probability that a mutation is somatic. The complexity of the cancer genome, including clonality and structural variation, coupled with the complex statistics of next-generation sequencing^14^ makes improving upon these statistical models challenging. Recently, state-of-the-art speed and accuracy have been achieved using machine learning for somatic variant calling with matched-normal samples^15–17^. Rather than attempting to model explicitly the likelihood functions for somatic mutations, these methods involve training a machine-learning classifier on a diverse training set with truth labels and applying the trained classifier to new oncology samples. Taking inspiration from these studies, we hypothesized supervised machine-learning algorithms would be effective for classifying mutations as somatic or germline in patient-derived solid tumor samples lacking a matched normal.

Tree-based machine-learning (ML) methods with gradient boosting—such as XGBoost^18^ and LightGBM^19^—consistently achieve high rank in open-science challenges such as Kaggle^20^ and DREAM^21^, and benefit from ease of implementation (e.g., the ability to handle missing and unnormalized data). Recently, Google released TabNet^22^, a deep-learning method designed for tabular input features and interpretability. Here we apply XGBoost, LightGBM, and TabNet to the problem of discriminating somatic and germline variants in WES oncology data, then compare the performance of these ML-based classifiers along with PureCN. All three ML-based methods achieve state-of-the-art performance with drastically reduced computational cost. We demonstrate the potential clinical utility of the ML-based classifiers by showing they eliminate the spurious TMB inflation associated with traditional tumor-only variant calling methods. This issue is most severe in racial minorities whose germline variants are underrepresented in commonly used variant databases^23^, yet we show ML classifiers are capable of fully removing this racial bias in tumor-only variant calling.

## Results

### Train/test overview

We used a somatic mutation calling pipeline to process samples both with and without the matched-normal sample (see “Methods”). Because TMB, copy-number variation (CNV), and sample composition (tumor purity) can impact somatic mutation calling, we selected oncology samples from different tissue types that span biological extremes, including ovarian adenocarcinoma (high purity, moderately low TMB, high CNV), STAD (low purity, low TMB), sarcoma (low TMB, high CNV), testicular germ cell cancer (extremely low TMB), endometrial carcinoma, colorectal adenocarcinoma, metastatic melanoma, and lung adenocarcinoma and squamous carcinoma (high TMB), and several other cancer subtypes from the Cancer Genome Atlas (TCGA). These subtype-related differences are based on analyses of the tumor samples and subtypes in this study, and all patient-level information, including subtype, TMB, purity, CNV burden, race, and ethnicity are included in Supplementary Table 1.

### Model

We engineered 30 mutation- and copy-number-specific features using tumor-only samples (see “Methods” and Supplementary Table 2). This included traditional features for somatic variant calling such as germline database frequency, COSMIC somatic mutation database^24^ counts, and read-based statistics such as variant allele fraction (VAF) and major allele frequency. These features are described in Supplementary Table 2. Expecting somatic mutations to exhibit a different mutational spectrum from germline variants, we also included features that characterize the trinucleotide context and base substitution subtypes that are the basis for mutational signature analysis^25^. The local copy number for each variant is represented by features derived from copy-number segmentation data and variant calls. Briefly, using germline variant databases and copy-number segments, we identify neighboring heterozygous germline SNPs of similar copy number, and create a histogram of variant counts with 20 non-overlapping VAF bins (see “Methods”).

The somatic and germline truth labels were determined by running an independent variant-calling pipeline using the matched-normal samples. Variants passing in the matched-normal pipeline were considered somatic; all other variants in the tumor-only pipeline were considered germline. The merged tumor-only feature matrix and truth labels were used for the binary somatic vs germline classification task.

**Fig. 1:**
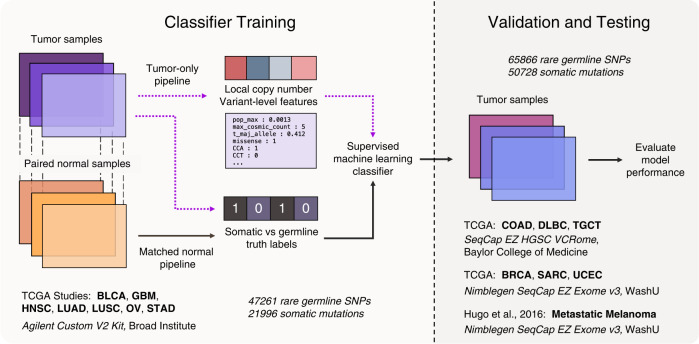
Machine-learning classifier for tumor-only somatic variant retrieval. To improve the reliability of tumor-only variant calling in whole-exome sequencing (WES) samples, we classify mutations as “somatic” or “germline” using TabNet, an attentive deep-learning classifier for tabular data, and gradient boosting tree-based methods XGBoost and LightGBM. We train models on solid tumor data in a supervised manner, using features from a tumor-only analysis and truth labels derived from a matched-normal analysis. We evaluate the trained model blindly on holdout tumor sample data that are biologically and technically distinct from the training set data—i.e., from different tissues of origin, exome-capture kits, and sequencing centers. Classifier training (left): To prepare the training set, we first align WES data for tumor and matched-normal samples from 105 oncology patients in The Cancer Genome Atlas across seven studies (BLCA bladder urothelial carcinoma, GBM glioblastoma multiforme, HNSC head and neck squamous cell carcinoma, LUAD lung adenocarcinoma, LUSC lung squamous cell carcinoma, OV ovarian serous cystadenocarcinoma, STAD stomach adenocarcinoma). For all patients, variant calling is performed with and without the matched-normal reference. CNV analysis is performed without the matched-normal samples. We extract features from the tumor-only variants and CNV data. The somatic or germline status of each variant detected in the matched-normal variant calling pipeline is used as the ground truth label—0 for germline and 1 for somatic. We combine the features and truth labels to train TabNet, XGBoost, and LightGBM classifiers to distinguish somatic from germline variants. Validation and testing (right): The model with the best average precision score on a validation set (COAD, DLBC, and TGCT, SeqCap EZ HGSC VCRome, Baylor College of Medicine) is selected and applied to two holdout test datasets of tumor-only samples: TCGA BRCA, SARC, and UCEC, and the metastatic melanoma dataset of ref. ^39^, using the Nimblegen SeqCap EZ Exome v3 kit. The accuracy of the tumor-only classification method is benchmarked using truth labels from the associated matched-normal pipeline.

To classify mutations, we selected three highly-performant machine-learning models for tabular data to classify mutations as somatic or germline: TabNet, an attentive deep-learning model^22^, XGBoost^18^, a gradient boosting tree-based algorithm, and LightGBM^19^, a similar tree-based model. TabNet leverages neural attention modules, and its feature masks, when visualized, allow interpretation for each classification instance, showing the saliency of each feature and each instance. TabNet has been shown to achieve state-of-the-art performance on tabular data, outperforming XGBoost and other powerful supervised machine-learning models, although results have been challenged by several studies. This study compares these three algorithms and PureCN in tumor-only variant calling. Figure 1 illustrates the overall train/validation/test scheme for the tabular machine-learning (ML) models.

### Training set construction

For our training set, we selected 105 tumor samples from distinct patients in seven cancer subtypes from TCGA (Supplementary Table 1). Somatic and germline truth labels were generated using the results of a variant-calling pipeline that included the patient-matched-normal samples. We engineered features for our tabular ML classifiers using the variant and CNV calls from the independent tumor-only pipeline, which used a process-matched normal blood sample panel (panel of normals) for each patient that, importantly, did not include the patient’s matched-normal sample (see “Methods”). The training dataset consisted of 15 samples from each of seven solid tumor cancer subtype studies in the Cancer Genome Atlas: bladder urothelial carcinoma (BLCA)^26^, glioblastoma multiforme (GBM)^27^, head and neck squamous cell Carcinoma (HNSC)^28^, lung adenocarcinoma (LUAD)^29^, lung squamous cell carcinoma (LUSC)^30^, ovarian serous cystadenocarcinoma(OV)^31^, and stomach adenocarcinoma (STAD)^32^. To enforce technical consistency within the training set, all the selected samples from these studies were sequenced at the Broad Institute using the Agilent Custom V2 exome-capture kit. This was done so we could later investigate whether our trained model could generalize to distinct exome-capture kits and subtypes in validation and test sets and still achieve high classification accuracy.

### Validation set construction

**Fig. 2:**
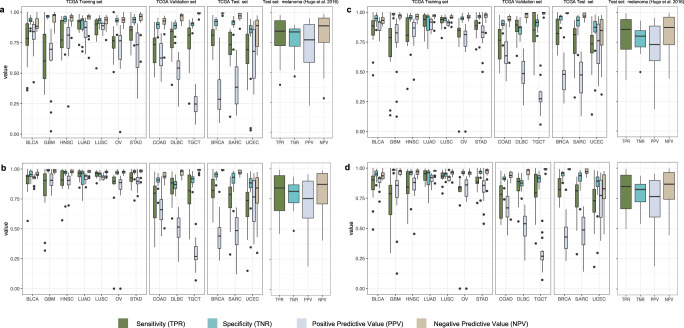
Patient-level performance of the classifiers across tissue types. Four accuracy metrics for TCGA datasets. *n* = 15 randomly selected cancer patients per tissue type. The tabular machine-learning (ML) classifiers were trained on BLCA, GBM, HNSC, LUAD, LUSC, OV, STAD studies, all with Agilent Custom V2 exome-capture kit (*n* = 105), validated on COAD, DLBC, and SARC studies, with SeqCap EZ HGSC VCRome capture kit (*n* = 45), and tested on BRCA, SARC, and UCEC Nimblegen SeqCap EZ v3 capture kit (*n* = 45) and the Hugo metastatic melanoma dataset (*n* = 23 cancer patients sequenced by UCLA). Predictions made with **a** TabNet, **b** XGBoost, **c** LightGBM, and **d** the ensemble average of models. Boxplots depict the median (center line), first and third quartiles (bounds of box), and maximum and minimum datapoints excluding outliers (whiskers). Outliers are datapoints more extreme than the box boundaries by a factor of 1.5 times the inner quartile range.

Our validation set consisted of 45 tumor samples from each of three cancer subtypes absent from the training data (15 samples each): colon adenocarcinoma (COAD)^33^, lymphoid neoplasm diffuse large B-cell lymphoma (DLBC), and testicular germ cell tumors (TGCT)^34^. These three TCGA cohorts were sequenced at Baylor College of Medicine with the SeqCap EZ HGSC VCRome capture kit. This WES kit has been shown to exhibit distinct genomic coverage compared to that of the Agilent Custom V2 kit^35^ used in training. We trained TabNet, XGBoost, and LightGBM on the training data and, in parallel, evaluated on the validation. This was done to ensure the parametrization of the models (see “Methods”) did not lead to overfitting on the training set. Strong performance on the validation set would suggest the trained model should generalize to new capture kits and tissue types. The resulting predictions on the training and validation sets were compared to the matched-normal truth labels and evaluated with further metrics (Fig. 2).

### Training and validation results

The optimally trained models fit the training data with AUCs of 0.96 (TabNet), 0.98 (LightGBM), and 0.99 (XGBoost). For metrics requiring binary values such as Matthews Correlation Coefficient (MCC) and sensitivity (true-positive rate, TPR), we used the training data to select the best posterior-probability threshold for somatic vs germline classification (see “Methods”). We also constructed an ensemble average, a simple average of the three models’ posterior probabilities for improved classification. Figure 2 displays patient-level performance for all models and TCGA datasets and tissue types. LUSC and LUAD have the best results across models, and as cancer subtypes with the highest TMB, they contribute strongly to the overall variant-level statistics from training. Despite the ease of fitting the training data, across all three models and the ensemble average, the training set sensitivity (true-positive rate, TPR) was consistently worst on GBM and OV subtypes, and the positive predictive value (PPV) consistently worst on GBM, OV, and STAD. This suggests that the challenge of identifying somatic mutations in the absence of a matched normal is highly tissue-specific.

The performance on the validation data was lower than the training set, with the optimally trained models achieving nearly identical AUC of 0.91–0.92, suggesting either slight overfitting of the model, or the validation data tumor types are more challenging to classify. In the validation set, TGCT exhibited high sensitivity and the lowest PPV. COAD exhibited the highest PPV and the lowest sensitivity. The reason for these tissue-specific differences is discussed below in “Explaining variability in performance”.

### Holdout test sets

After model training and selection, we constructed two separate holdout test sets, including four cancer subtypes and a new exome-capture kit, Roche Nimblegen SeqCap EZ Exome v3. Model results on these blind holdout test sets are shown in Table 1 and Fig. 2.

**Table 1:** Model performance metrics on blind holdout test sets.

Dataset	Variant category	Method	AUC	MCC	TPR	TNR	PPV	NPV	Balanced accuracy	FP	TP	FN	TN	Call rate
Blind test set— BRCA SARC UCEC	Overall: 30,270 somatic, 31,359 germline	TabNet	0.942	0.762	**0.931**	0.828	0.839	**0.926**	0.879	5397	28,184	2086	25,962	100
		XGBoost	0.946	0.757	0.865	0.892	0.885	0.873	0.878	3392	26,186	4084	27,967	100
		LightGBM	**0.949**	**0.766**	0.874	0.892	**0.886**	0.88	**0.883**	3399	26,451	3819	27,960	100
		PureCN	0.85	0.592	0.662	**0.912**	0.882	0.729	0.787	2237	16,792	8588	23,065	82.2
	SNVs: 29,593 somatic, 30,122 germline	TabNet	0.945	0.775	**0.943**	0.827	0.843	**0.937**	0.885	5199	27,918	1675	24,923	100
		XGBoost	0.949	0.77	0.879	0.891	0.888	0.882	0.885	3286	26,006	3587	26,836	100
		LightGBM	**0.951**	**0.776**	0.884	0.892	**0.889**	0.887	**0.888**	3268	26,159	3434	26,854	100
		PureCN	0.851	0.593	0.662	**0.913**	0.887	0.724	0.788	2082	16,343	8360	21,983	81.7
	Indels: 839 somatic, 1762 germline	TabNet	0.784	0.433	**0.838**	0.624	0.515	**0.89**	0.731	662	703	136	1100	100
		XGBoost	0.799	0.408	0.666	0.757	0.566	0.827	0.712	428	559	280	1334	100
		LightGBM	0.815	0.455	0.765	0.718	0.564	0.865	0.742	497	642	197	1265	100
		PureCN	**0.827**	**0.529**	0.606	**0.893**	**0.756**	0.805	**0.749**	132	410	267	1105	73.6
Blind test set —metastatic melanoma	Overall: 15,813 somatic, 12,871 germline	TabNet	0.852	0.55	0.797	0.753	0.799	0.751	0.775	3176	12,598	3215	9695	100
		XGBoost	0.861	0.558	0.801	**0.756**	**0.802**	0.756	0.779	3135	12,670	3143	9736	100
		LightGBM	**0.867**	**0.57**	**0.823**	0.744	0.798	**0.774**	**0.784**	3289	13,020	2793	9582	100
		PureCN	0.824	0.52	0.789	0.732	0.794	0.726	0.76	2951	11,340	3041	8050	88.5
	SNVs: 15,688 somatic, 12,412 germline	TabNet	0.85	0.545	0.796	**0.749**	0.8	0.744	0.773	3114	12,490	3198	9298	100
		XGBoost	0.862	0.565	0.815	0.748	**0.804**	0.762	0.782	3125	12,788	2900	9287	100
		LightGBM	**0.868**	**0.573**	**0.833**	0.737	0.8	**0.777**	**0.785**	3266	13,061	2627	9146	100
		PureCN	0.823	0.516	0.788	0.729	0.797	0.717	0.758	2856	11,228	3028	7686	88.2
	Indels: 147 somatic, 611 germline	TabNet	0.82	0.361	0.837	0.619	0.346	0.94	0.728	233	123	24	378	100
		XGBoost	0.833	0.384	0.837	0.646	0.363	0.943	0.742	216	123	24	395	100
		LightGBM	0.84	0.343	**0.898**	0.534	0.317	0.956	0.716	285	132	15	326	100
		PureCN	**0.887**	**0.624**	0.888	**0.826**	**0.581**	**0.964**	**0.857**	80	111	14	379	77

*AUC* area under the receiver operating characteristic curve, *MCC* Matthews Correlation Coefficient, *TP* true positives— somatic mutations correctly classified as somatic, *FP* false positives—rare germline variants misclassified as somatic mutations, *FN* false negatives—somatic mutations misclassified as germline variants, *TN* true negatives—rare germline mutations correctly classified as germline. Bold values indicate the best performance for a given metric, variant category, and test set.

Benchmark accuracy metrics for tumor-only somatic vs germline classification by TabNet, XGBoost, LightGBM, and PureCN on blind test datasets. Overall performance considers all single-nucleotide variants (SNVs) and indels.

Call rate—percentage of total coding variants classified.

The first holdout test set included solid tumor samples from 45 patients from the following three TCGA studies (15 each): breast invasive carcinoma (BRCA)^36^, sarcoma (SARC)^37^, and uterine corpus endometrial carcinoma (UCEC)^38^. These samples were sequenced at Washington University in St. Louis. Table 1 displays the trained model’s performance on the holdout test datasets. Grouping SNVs and indels together, LightGBM achieved the best AUC (0.949), MCC (0.766), PPV (0.886), and balanced accuracy (0.883). TabNet had the best TPR and negative predictive value (NPV). All models perform better on SNVs than indels, and PureCN showed particularly strong specificity (true-negative rate, TNR) for indels. The subtype associated with the highest PPV across all models was UCEC and the highest TPR, BRCA. Symmetrically, UCEC had the lowest TPR and BRCA the lowest PPV for all models. These results suggest that the biological difference between cancer tissue subtypes is more influential than the choice of the machine-learning model on performance.

To validate further the generalization of the models and their robustness to batch effects, we acquired a final holdout dataset comprised of non-TCGA data. This final holdout dataset included 23 samples from the Hugo et al., 2016 metastatic melanoma study^39^. Relative to the TCGA test set, our model performed better in both TPR and PPV on this dataset at the patient level (Fig. 2b). Yet performance on the overall variant level was slightly lower due to the influence of high-TMB patients. Variant-level AUC values (indels and SNVs) in the melanoma dataset were 0.85 (TabNet), 0.86 (XGBoost), and 0.87 (LightGBM). Again, LightGBM slightly outperformed the other models in AUC, MCC, and balanced accuracy.

### Concordance of tumor-mutational burden estimation methods

The reliable estimation of TMB is a critical benchmark for a model designed to improve tumor-only variant calling. This capacity is especially relevant in immuno-oncology clinical trials where TMB is a strong biomarker of response^40^ and survival^1^ and where matched-normal samples are not always available. We define our naive tumor-only method as a non-machine-learning approach that incorporates a process-matched panel of normals, multiple germline variant databases, and standard variant filtering techniques (see “Methods”) to remove germline variants and artifacts. Our machine-learning-based approach applies the tabular models’ somatic vs germline classifications to the results of the naive approach. For simplicity, we use a somatic posterior-probability cutoff of 0.5 to isolate predicted somatic mutations for refining estimates TMB.

**Fig. 3:**
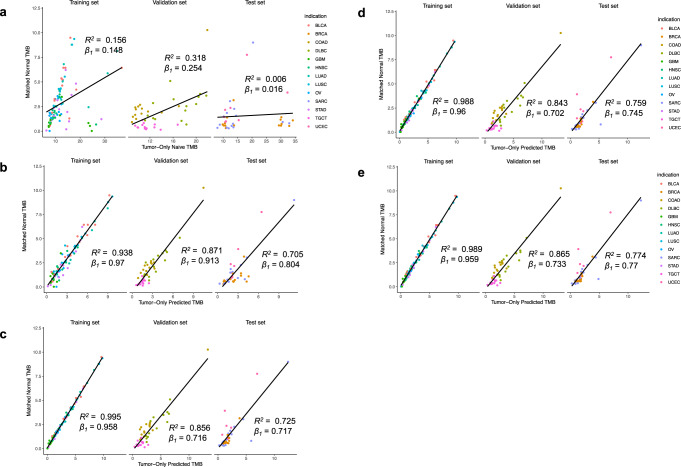
Concordance of tumor-mutational burden calculated with and without matched normals. Training set, *n* = 105; Validation set, n = 45; Test set, *n* = 45 patients. **a** Matched-normal TMB compared to TMB estimated by the naive tumor-only approach—variants are filtered by removing common germline variants using multiple population germline databases and a process-matched leave-one-out panel of normals. **b**–**e** Matched-normal (”true”) TMB compared to TMB estimated with somatic mutation classification via **b** TabNet, **c** XGBoost, **d** LightGBM, and **e** ensemble average of all three models *β*_*1*_ indicates the slope of linear regression fit.

To evaluate reliability, we compared the naive and machine-learning-based TMB estimates with those derived from the matched normal “gold standard” (Fig. 3). Using linear regression, we calculated an *R*^2^ of 0.156, 0.318, and 0.006 for TCGA train, validation, and test sets, respectively, indicating a weak correlation between matched and tumor-only TMBs (Fig. 3a). Notably, in the BRCA, SARC, and UCEC test set, the rank order of TMBs is markedly different between naive and matched-normal methods. The slopes of these fits (0.148, 0.254, 0.016 for TCGA training, validation, and test datasets, respectively) are substantially less than 1.0 in all cohorts, indicating a consistently inflated TMB result for tumor-only samples. The magnitude of this inflation agrees with recently reported results^6,7^.

Next, we evaluated the relationship between TMB from our tumor-only somatic predictions of the tabular ML models to the TMB from our matched-normal variant calling pipeline (Fig. 3b–e). Linear regression fits to the test set yielded *R*^2^ values of 0.705 (TabNet), 0.725 (XGBoost), 0.759 (LightGBM), 0.774 (ensemble average), indicating a 117–129-fold improvement over the naive method (Fig. 3). The slope of best fit was similarly encouraging on the test set, 0.804 (TabNet), 0.717 (XGBoost), 0.745 (LightGBM), 0.770 (ensemble average) with our model achieving a 45–50-fold improvement relative to the naive approach. The improvement offered by these tabular models argues for the use of ML-corrected TMB estimates for clinical variant analysis in tumor-only samples.

### Eliminating the impact of racially biased germline databases in tumor-only variant calling

**Fig. 4:**
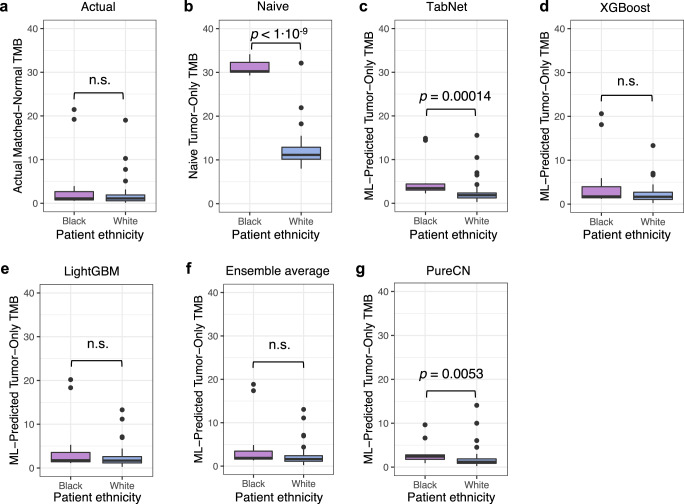
Impact of racial bias in germline databases on tumor-mutational burden (TMB) estimates in tumor-only WES samples. Each panel displays patients from TCGA validation and holdout test sets, *n* = 12 Black and 55 white patients. **a** True TMB from matched-normal pipeline. **b** TMB estimates without matched-normal samples, using multiple germline population databases and a process-matched leave-one-out panel of normals (naive tumor-only method). **c**–**g** Corrected tumor-only TMB estimates using **c** TabNet, **d** XGBoost, **e** LightGBM, **f** ensemble average of tabular machine-learning (ML) models, and **g** PureCN. Boxplots depict the median (center line), first and third quartiles (bounds of box), and maximum and minimum datapoints, excluding outliers (whiskers). Outliers are datapoints more extreme than the box boundaries by a factor of 1.5 times the inner quartile range.

The underrepresentation of racial minorities in genomic databases has widespread negative consequences in human genome science and has been the subject of intense criticism^41,42^. Tumor-only variant calling is no exception^43^. A recent study observed that the inflation of TMB caused by the absence of a matched-normal sample is most severe in underrepresented minorities^23^. Comparing the matched-normal TMBs of the 12 Black patients and 55 white patients in the TCGA validation set and holdout test set, we see no statistical difference in this “true” TMB between the two groups (*p* > 0.05, Wilcoxon test) (Fig. 4a). In the absence of a matched-normal sample, however, the difference is profound (*p* « 0.001) with median tumor-only TMBs of Black patients (30.36) being almost three times as high as that for white patients (11.15) (Fig. 4b).

After applying the predictions of the tabular models to eliminate rare germline variants, the inflation of TMB for Black patients is greatly reduced and low like that of white patients. For XGBoost (Fig. 4d) and LightGBM (Fig. 4e), the *p*-value is not significant, suggesting full elimination of bias. For LightGBM, median TMBs are 1.76 for Black patients and 1.68 for white patients, a biomarker difference so small that it would be highly unlikely to make a difference in clinical trial enrollment. For TabNet (Fig. 4c), the corrected median TMBs for Black and white patients are 3.43 and 1.85, respectively, and PureCN (Fig. 4g), 2.41 and 1.22. So TabNet and PureCN are still slightly biased by 1–1.5 mutations per megabase (significant in this cohort with relatively few Black patients), yet all these algorithmic methods constitute a dramatic improvement relative to the TMB inflation of ~19 mutations per megabase seen with the naive tumor-only method (Fig. 4a).

### Comparison to PureCN

**Fig. 5:**
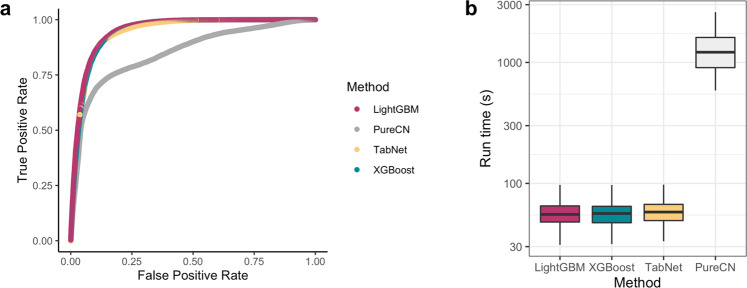
Benchmark for accuracy and compute times for PureCN, TabNet, XGBoost, and LightGBM on TCGA holdout test data. **a** Receiver operating characteristic (ROC) curve calculated for PureCN and tabular machine-learning models, treating somatic mutations as positives and germline variants as negatives. Curves display 500 distinct posterior-probability thresholds for classification, selected by binning the probabilities into 500 quantiles. **b** Run-time comparison in seconds. (PureCN, 250 CPUs per sample; XGBoost and LightGBM, 1 CPU per sample; TabNet 1 CPU per sample, no GPU). Boxplots depict the median (center line), first and third quartiles (bounds of box), and maximum and minimum datapoints, excluding outliers (whiskers). Outliers are datapoints more extreme than the box boundaries by a factor of 1.5 times the inner quartile range.

By varying the posterior-probability threshold across 500 quantiles, we constructed ROC and precision-recall curves for TabNet, XGBoost, LightGBM, and PureCN. Figure 5a displays the ROC curve comparing TabNet and PureCN for the BRCA, SARC, and UCEC TCGA holdout test set of 45 patient samples. All algorithms are highly tunable. The tabular ML models have higher AUC and are consistently concave down, suggesting more stable dependence on posterior-probability cutoffs.

For the holdout datasets, tabular ML models achieved better overall performance and better performance on SNVs, yet PureCN achieved better performance on indels (Table 1). On the TCGA holdout test set, all tabular models see a >9% improvement in AUC and >16% improvement in MCC over PureCN. For indels, PureCN outperformed all tabular models by >7% MCC with marginal gains to AUC. On the second holdout test set consisting of 23 metastatic melanoma patients, tabular models perform similarly to PureCN, both overall and for SNVs (tabular models are 3–4% better AUC). Indel AUCs are comparable, but PureCN’s indel MCC is substantially better by >24.4% MCC, with 205 fewer false positives than TabNet and only 21 fewer true positives.

Next, we compared the amount of time elapsed to make somatic/germline predictions starting from annotated tumor-only VCFs. This includes feature engineering and model inference but not model training. The compute time of LightGBM (mean 55.4 s) on a single core was 21.9 times faster than PureCN’s (1214.2 s, *P*«0.001) using 250 cores (Fig. 5b). This dramatic compute speed improvement over PureCN is not surprising as trained supervised machine-learning classifiers are known to have a less intense CPU requirement than Bayesian methods.

### Global feature importance

**Fig. 6:**
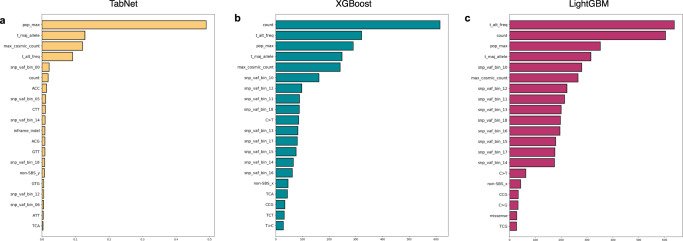
Feature importance for trained tabular machine learning somatic vs germline classifiers. Top 20 most important features shown for **a** TabNet, **b** XGBoost, and **c** LightGBM. *pop_max*, maximum population frequency of variant across multiple germline databases; *t_maj_allele*, the fraction of reads containing the most supported allele at that locus in the sample; *max_cosmic_count*, the number of occurrences of the variant in COSMIC somatic database; *t_alt_freq*, the fraction of reads supporting the alternate allele; *snp_vaf_bin_i*, the number of informative SNPs (heterozygous SNPs common in germline databases whose VAF should inform the local copy number) in the copy-number segment with a VAF between i/20 and (i + 1)/20 where *i* is an integer between 0 and 19; *count*, the total number of variants to classify in that sample; *ACC*, *CTT*, *etc*, trinucleotide context; *non-SBS-y*, non-single-base substitution (meaning no trinucleotide context applies). The full list of 30 features and brief descriptions are included in Supplementary Table 2.

We inspected the global feature importances of our trained classifiers. The top 20 out of 56 total features for the three tabular ML models are shown in Fig. 6. The maximum population allele frequency across multiple germline databases (*pop_max*) is the most important feature for TabNet (Fig. 6a). Interestingly, in XGBoost (Fig. 6b) and LightGBM (Fig. 6c), the most important feature is *count* (the total number of variants to classify in the sample) rather than *pop_max*, which appears as third most important. The lower dependency on population databases likely underlies the elimination of racially biased TMB inflation in these models. Or perhaps knowing *count*, the total number of mutations to classify—which depends largely on the number of rare germline variants absent from the biased databases—allows LightGBM and XGBoost to recognize and make better decisions with samples from patients in underrepresented groups. Other important features for all models are *t_maj_allele* (the greatest VAF among all observed alleles at the variant’s locus), *max_cosmic_count* (the number of times the variant is observed in COSMIC, the catalog of somatic mutations in cancer^24^, *t_alt_freq* (the VAF of the mutant allele), snp_vaf_bin_00 (the number of neighboring heterozygous common germline SNPs with VAF between 0.0 and 0.05), and the set of snp_vaf_bin features corresponding to VAFs between 0.5 and 0.65. *inframe_indel* in TabNet and *missense* in LightGBM are the only ontology-related feature in the top 20. The remaining features in the top 20 are from either the *snp_vaf_bin* class of features or are related to the mutational spectrum.

Together, these features are reasonably ordered, with *count* or *pop_max* being the most important, and allele fraction and COSMIC features near the top. A surprise is the low feature importance of the ontology features, which include reading-frame mutation consequences, such as *nonsense* or *missense*. This was unusual because nonsense mutations are expected to occur more commonly as somatic mutations than as germline variants. Indeed, 63% of the nonsense variants in our test set are truly somatic, whereas for all variants in our test set, the truth labels are 49% somatic and 51% germline. The ontology features including *missense, nonsense*, and *inframe_indel* together add up to low importance. But otherwise, the feature importances for the three models appear to be well-ordered.

### Explaining variability in performance

Using multiple regression models, we explain individual performance as a function of several predictor variables. We observed that the most influential factor on a sample’s positive predictive value (PPV) is the “true” TMB coming from germline subtraction via the matched-normal pipeline. Samples with lower TMB tend to have a lower PPV (*R*^2^ = 0.54, Supplementary Fig. 2), so a paucity of true somatic mutations in a sample contributes strongly to a low PPV in ML-based tumor-only variant classification. Though highly predictive of model performance, the true TMB is only estimated without a matched-normal sample. We suspect that a small yet consistent number of germline variants appear somatic based on low variant allele fraction (VAF) leading to a consistent number of false positives. Supporting this, the median VAF of false positive (FP) and true negative variants (TN) was 0.35, and 0.50, respectively. A larger panel of normals, including more rare germline variants, would mitigate this effect, yet perhaps with diminishing returns.

The interpretable feature masks of the TabNet architecture allow us to explain explicitly which features contribute most to these false positive calls. By examining the feature masks, which indicate where the TabNet neural network distributes its attention for each classification instance, we see surprisingly that the VAF (*t_alt_freq*) is not the most distinguishing feature between FPs and TNs. Rather, it is the COSMIC count (*max_cosmic_count*, the number of times the variant is observed in COSMIC) and the overall count of mutations (*count* variable) that best distinguish somatic and germline predictions, with differences being present in the first feature mask layer (Supplementary Fig. 3). Illustrating the explanatory utility of these feature masks, we found the proportion of variants with a nonzero *max_cosmic_count* was significantly greater for FPs than for TNs (*P* < 0.00001, Fisher’s exact test), with 993 out of 5397 for FPs (18%) and 3612 out of 25962 (14%) for TNs, and further, the mean *max_cosmic_count* values was lower for FPs (0.99) than for TNs (0.26), *P*«0.001 (Wilcoxon rank-sums test). We also found that the number of mutations to classify (*count* variable, rare germline SNPs + true somatic mutations in the sample) was greater in FPs (3390) than for TNs (2287), *P* ≪ 0.001. Thus, significant differences are found between correctly and incorrectly classified germline variants for both features. Together this exemplifies how the feature masks of TabNet help with interpreting classifications.

Considering FPs, TNs, alongside true positives (TP) and false negatives (FN), as shown in Supplementary Fig. 3, the attention of the TabNet classifier is consistently applied to *max_cosmic_count* for all mutations classified as somatic (TP + FP), and to *count* for all variants classified as germline (TN + FN). Limited interpretability is a common and valid criticism of non-attention-based deep-learning models, but attention-derived insights such as those presented here and by others^44^ offer a way to interrogate deep-learning models and avoid reliance on predictions from “black box” neural networks.

The sensitivity (TPR) of our models is best explained by the median VAF of the true somatic mutations (MVTSM), a value that can be interpreted as an approximation for tumor purity divided by 2 (Supplementary Fig. 4), which remains unknown without a matched normal. The regression model for TPR with MVTSM as the sole predictor has the following parameters: *R*^*2*^ = 0.4, *β*_*0*_(*y*-intercept) = 0.99, *β*_*1*_(slope) = −0.89. Thus, the following approximation predicts the TPR of our model:

$${{{\mathrm{TPR}}}} \approx 1 - {{{\mathrm{MVTSM}}}}.$$

Another interpretation is that the TPR increases with increasing stromal fraction, the so-called “contaminating normal tissue” in the biopsy. Covariance analysis of TPR vs MVTSM across indications identified GBM, melanoma, and BLCA as the tissue types in our datasets where this relationship is strongest (Supplementary Fig. 5).

## Discussion

We constructed and trained three types of tabular machine-learning (ML) classifiers to distinguish somatic mutations from rare germline variants. Our models were trained on seven cancer subtypes sequenced by the Broad Institute with a single exome-capture kit. The trained models successfully generalize to two distinct capture kits and seven distinct cancer subtypes in the validation and two blind holdout sets. For accurate predictions, the cancer subtype differences appear to vastly outweigh technical NGS variability and model choice. Tabular ML models outperform PureCN in both speed and overall accuracy. The ML predictions confer agreement between matched and tumor-only TMB calculation, with a substantial fold improvement over the naive method and a slope within 20% of 1.0, enabling reliable somatic mutation retrieval in tumor-only variant calling and harmonization of TMB calculation in cohorts of mixed tumor-only and matched-normal WES samples.

The performance metrics reported in this study are for models trained on seven cancer subtypes and a single exome-capture kit and sequencing center. We expect a model trained on more diverse input data would generalize better than ours, and we expect our reported performance to increase with training set size and diversity. Training on a dataset with tumors of different subtypes, purity, ploidy, copy-number profiles, and mutational spectra, as well as multiple WES data sources will likely improve the performance of a model applied to a cohort of contrasting biology and technical data quality. Conversely, analyzing a homogenous cohort (e.g., from a single cancer subtype in a clinical trial) may benefit from training on a similar cohort, especially if including subtype-dependent features like nucleotide substitution types. In cohorts with a mix of matched-normal and tumor-only samples, it is straightforward to estimate the performance on the tumor-only subset in a way akin to the methodology outlined in this work, by running parallel matched-normal and tumor-only variant-calling pipelines on the matched-normal subset and evaluating the resulting classifications with the matched-normal truth labels.

The tree-based XGBoost and LightGBM methods slightly outperformed TabNet, despite the promising claims by the authors of TabNet. The addition of TabNet did, however, make the ensemble average predictions modestly better, suggesting the best single model does depend on the classification instance. These model-specific results largely recapitulate recent comparisons between tree-based methods, TabNet, and other deep-learning models for tabular data^45,46^. In addition to the slightly better overall accuracy of XGBoost and LightGBM, in this study, the tree-based models excelled at reducing racial bias associated with tumor-only variant calling. The feature importances shed light on why this is the case: the most important classification features for both tree models were unrelated to germline databases, whereas TabNet highly prioritized a feature encoding inclusion across germline databases. A unique feature of TabNet is that TabNet’s feature masks enable out-of-the-box interpretation of individual classification results. This is not as straightforward with non-attentive deep-learning methods^47^ nor the tree methods we tested. The extent to which attentive models offer faithful explanation of predictions has been debated, working in some contexts and not others^45,46^, but in this work, we see concordance between the values in the feature masks and statistical differences in the data.

For somatic mutation calling with DNA sequencing data, we expect no algorithm will ever be as good as having the matched normal. One can imagine a perfectly clonal, diploid tumor without copy-number alterations and without any contaminating normal tissue (100% purity). In this hypothetical tumor, the variant allele fraction of somatic mutations would be distributed identically to the germline variants. Somatic mutations may exhibit characteristic genomic distribution and nucleotide substitution patterns^48,49^, offering a modest advantage for somatic vs germline classification in some tumor types. Yet, without a substantial fraction of normal stromal tissue in the bulk WES biopsy, we expect methods based partly on VAF statistics such as ours will never perform as well as having the matched-normal sample.

A major limitation in human genomics and precision medicine is that not all subpopulations are well-represented in genomic studies^41^. Human germline variant databases predominantly consist of subjects of white European ancestry, and this bias fragments the reliability of naive tumor-only variant calling methods for Black patients more so than white patients^23,43^. By integrating multiple informative features such as the total number of variants to classify (somatic + rare germline SNPs), COSMIC and germline databases, variant allele fractions, and the local copy-number ratios of known heterozygous germline SNPs, we were able to substantially reduce the racially biased overestimation of tumor-only TMB with TabNet and PureCN, and eliminate this bias below the practical limit with XGBoost and LightGBM.

## Methods

### TCGA genomic data acquisition

Manifest files for downloading TCGA genomics data were generated using the TCGA-Biolinks^50^ R package. 15 patients for each indication were selected from TCGA randomly, provided the patient had a single tumor sample and a single normal sample. 15 acute myeloid leukemia samples were originally included in the training set but were removed due to the presence of somatic mutations in the normal samples. BAMs were downloaded from GDC using the GDC Data Transfer tool. The samtools “collate” command was used prior to extracting FASTQs from the GDC BAMs with samtools “bam2fq”. Capture-kit information (including name, vendor, and catalog number) for each sample was queried using the GenomicDataCommons R Package, and these data are included in Supplementary Table 1. Patient ethnicity information was acquired from the clinical information on the TCGA data portal (https://portal.gdc.cancer.gov/). Ethnicity was self-identified by the patients in these studies, and categories included white, Black, Asian, American Indian/Alaska Native, and Native Hawaiian/Other Pacific Islander.

### Hugo et al. metastatic melanoma WES data acquisition

Sequencing data from 23 metastatic melanoma patients sequenced at UCLA were downloaded from SRA using SRA toolkit and the command **“**fastq-dump -split-3 –gzip $SRR”. The 23-sample subset was chosen because it had available capture kit metadata.

### Alignment

FASTQs were aligned to hg38 using the Sentieon implementation of BWA-MEM^51^. We used a consistent bioinformatics approach across all batches and cohorts.

### Panel of normals construction

A panel of blood or tissue normal samples sequenced under a common NGS protocol (panel of normals) is routinely used in whole-exome sequencing analysis to filter out germline SNPs and alignment and technical artifacts inherent to the capture-kit choice. It is also used for CNV analysis—the germline copy number of many samples are used to average or represent the capture-kit-specific depth biases so that biological copy-number variation can be isolated. A leave-one-out panel of normals strategy was chosen to maximize the number of normal samples available for training. A further benefit of the method is it ensures that the racial demographics of the normal samples in the panel are representative of the cohorts used in training and evaluation.

A separate leave-one-out panel of normals was constructed for each of the 195 TCGA patients in this study. For a given capture kit with *N* patients sequenced, the leave-one-out approach is as follows: for each of the *N* patients, gather the *N* − 1 normal samples from every other patient, and use these *N* − 1 normal samples to create both the CNV log_2_-copy-number reference and normal panel VCF (VCF panel of normals). This strategy is analogous to leave-one-out cross-validation. The CNV and VCF panel of normals from TCGA data were matched with the capture kit of the tumor samples. For the 23 metastatic melanoma samples, CNV and VCF normal panels were both derived from a randomly chosen patient from the Nimblegen SeqCap EZ Exome v3 sequenced TCGA cohort.

### Variant-level panel of normals construction

BCFtools^52^ and the “merge” command were used to aggregate the germline VCFs of the *N* − 1 normal samples. All identified variants occurring in at least two of the samples were added to the normal panel VCF.

### Copy-number panel of normals construction

CNVkit^53^ generated .cnn files that were aggregated to a panel of normals using the CNVkit “reference” command. The bins were specified using the capture kit’s baits BED file, lifted over from hg19 to hg38 with the UCSC LiftOver tool.

### Copy-number calling

We used CNVkit to generate log2 copy-number ratios and segments using the circular binary segmentation algorithm. For single TCGA samples, their associated leave-one-out panel of normals was used with the CNVkit batch mode. For the Hugo melanoma cohort, batch mode was also used, with a CNV panel of normals from a randomly chosen TCGA patient’s leave-one-out panel of normals.

cnvkit.py batch $tumor -r $pon -p $procs_per_job --output-dir $sample

cnvkit.py call $sample/$sample\_tumor.cns -o $sample/$sample.call.cns

For each tumor sample, we calculated two metrics for patient-level CNV alteration--CNV burden and CNV segment count (Supplementary Table 1). CNV burden is calculated as the fraction of the genome (in base pairs) that is altered (non-diploid) as inferred using the CNVkit ‘call’ command. The segment count is the total number of copy-number segments in the CNV results, derived from circular binary segmentation as implemented in CNVkit.

### Variant calling

Sentieon’s TNScope^54^ was applied to the hg38-aligned BAMs and the capture-kit-matched panel of normals. No patient-matched normals were included in the process-matched panel of normals. SnpSift v4.3 added dbSNP^55^ build 151 and COSMIC^24^ v85 annotations to all VCFs with the following command:

SnpSift Annotate -a $COSMIC_VCF $SNP_EFF_ANNOTATED_VCF

dbNSFP4.0^56^ was used to annotate variants with databases such as 1000 Genomes^57^ and ExAC^58^. We constructed “pop_max”, a single-aggregate feature derived from dbNSFP for filtering and the machine-learning model. pop_max, calculated by taking the maximum population allele frequency across the following dbNSFP databases: 1000Gp3_AF, TWINSUK_AF, ALSPAC_AF, UK10K_AF,ExAC_AC,ExAC_AF,gnomAD_exomes_AF, gnomAD_genomes_AF.

### Variant filtering

A set of criteria was chosen for pre-filtering variants such that artifacts and common germline SNPs are eliminated before applying training or applying the tumor-only classifier. These eliminated variants do not count as true negatives; thus, our specificity and NPV metrics are calculated conservatively. The criteria isolated passing, coding mutations for all tumor-only variant calls and is as follows: population allele frequency <0.01 across the 8 germline databases, SnpEff annotation ontology in missense, nonsense, frameshift_indel, or inframe_indel, FPfilter == “PASS”, Sentieon TNScope filter == “PASS”.

FPfilter^59^ eliminated sequencing and alignment artifacts. TNScope filter flags likely sequencing errors (using the *t_lod_fstar* variable based on Mutect2) as well as artifacts and germline mutations identified with the process-matched panel of normals. We discarded these variants and kept only the variants that we’d consider to be somatic coding mutations.

### PureCN

We ran PureCN using the production configuration recommended in the official documentation. For input, we used the COSMIC and dbSNP-annotated tumor-only VCFs after removing artifacts from the VCFs using bcftools (TNScope filter == “PASS”). A normalDB was constructed for every PoN VCF used in this study with the command Rscript $PURECN /NormalDB.R --outdir $out_dir --normal_panel $pon_vcf --assay $patient_id + --genome hg38 –force.

The copy-number ratio.cnr files from CNVkit were converted to segmentation files (.seg) using the CNVkit ‘export seg‘ command. The hg38_simple_repeats.bed file was downloaded from UCSC to blacklist SNPs in tandem repeat regions^60^. 250 cores were used per sample and the “—postoptimize” flag was turned on. The full command is as follows:

Rscript $PURECN --version; Rscript $PURECN --out $out_dir --sampleid $patient_id --tumor $COPY_NUMBER_RATIO --segfile $seg_file --mappingbiasfile $normal_db --vcf $vcf --snpblacklist $simple_repeats --genome hg38 --parallel --cores 250 --funsegmentation Hclust --force --postoptimize --seed 123.

### Comparing models to PureCN

Unfiltered variants from our variant calling pipeline were merged with the classified variants from PureCN. Variants were subsequently filtered using the same criteria and thresholds that we applied to isolate coding somatic mutations, including the TNScope filter^54^, FPfilter^59^, coding mutation ontology, and population database frequency. TabNet, XGBoost, and LightGBM predictions were merged, and call rate was assessed for TabNet and PureCN by calculating the number of variants with posterior somatic probability predictions. True positives were defined as somatic mutations correctly classified as somatic; false positives, rare germline variants misclassified as somatic; false negatives, true somatic mutations misclassified as germline; true negatives, rare germline variants correctly classified as germline.

### Tree-based model training

Python modules XGBoost version 1.2.1 and LightGBM version 3.3.2 were used for the tree-based classifiers. Default parameters were used for XGBoost. The following hyperparameters were specified for LightGBM: *objective*: “binary”, *num_iteration*: 10000, *num_leaves*: 30, *learning_rate*: 0.1, *bagging_fraction*: 0.7, *feature_fraction*: 0.7, *bagging_frequency*: 5, *bagging_seed*: 2018, *verbosity*: −1.

### TabNet training

For running TabNet, we used the open-source PyTorch implementation (https://github.com/dreamquark-ai/tabnet) with PyTorch version 1.7.0. The following model hyperparameters were used to build the TabNet architecture: n_d = 24, n_a = 24, n_steps = 4, gamma = 1.5, n_independent 2, n_shared = 2, lambda_sparse = 0.0001, momentum = 0.3, clip_value = 2.

Training was achieved over 100 epochs, using the Adam Optimizer with a learning rate of 0.02, a patience of 100, a batch size of 4000, and a virtual batch size of 256. Although TabNet does not require categorical features to be one-hot-encoded, we did this to allow for more flexibility with the other machine-learning models. A custom loss function was designed to maximize the average precision score (the area under the precision-recall curve). We trained the model for a total of 100 epochs, after which we selected the model from the epoch with the best performance on the validation set. We repeated the train-validate-test process three times to ensure the reproducibility of this training strategy. Training completed in less than 2 h on the CPU, and approximately 15 min using GPU acceleration with an NVIDIA P100. GPUs were not used for the time benchmark comparing TabNet predictions to PureCN.

### Posterior-probability thresholds for binary metrics

After inspecting performance in the three categories (overall, SNVs, and indels), we noticed different probability thresholds yielded optimal results for the different variant categories, for both tabular ML models and PureCN. For example, for SNVs, TabNet’s optimal F1-score occurred at a cutoff of 0.508, PureCN’s at 0.005. For indels, the optimal F1-score occurred at a threshold of 0.1368 for TabNet and 0.014 for PureCN. We used these probability thresholds derived from performance on the training set to make our binary predictions for the blind test sets. Those results are reported in Table 1 and Fig. 2.

### Regression and covariance analysis

Linear regression and covariance calculations were calculated using the R computing environment, version 3.5.2. To calculate quartiles in Supplementary Fig. 4a, we used bootstrap sampling with 1000 bootstrap replicates.

### TMB calculation

There is no consensus on how to normalize TMB in whole exome sequencing, i.e., whether to use the size of the exome in the human genome or the size of the regions targeted by the exon capture kit. Often the TMB is presented without normalizing. We obtained exon target.BED files for the three capture kits in this study from the manufacturer’s websites. We used the UCSC liftOver tool to convert them from hg19 to hg38. The total footprint of the exon targets from SeqCap EZ HGSC VCRome, and Nimblegen SeqCap EZ Exome v3 kits, was 33.0, 37.3, and 63.5 MB, respectively. Since we used three distinct exon capture kits in this study, for simplicity, we decided to normalize the total somatic mutation count across all datasets by dividing by a constant factor: 41, corresponding to the patient-weighted average of the three kits’ target footprint size in megabases.

### Reporting summary

Further information on research design is available in the Nature Research Reporting Summary linked to this article.

## Supplementary information

Supplementary Material

REPORTING SUMMARY

Supplementary Table 1

Supplementary Table 2

## Data Availability

All genomic and clinical data used in this study are available online. Controlled-access TCGA whole-exome sequencing data were acquired through Genomic Data Commons. This required authorization from dbGaP via individual researcher and institution registration on NIH eRA commons, and a written description of the research project requiring access to controlled data. All TCGA filenames and sample IDs used in this study are provided in Supplementary Table 1. Metastatic melanoma whole-exome sequencing data were acquired from SRA under the accession number SRP067938. All processed data generated for this study are available from the authors upon request.

## Code availability

The tumor-only somatic-germline classifier described in this study was written in Python. All code including feature engineering and model training and evaluation is available at https://github.com/AbbVie-GRC-Methods-Dev/new_normal and is usable under the MIT license. The following software and versions were used: Sentieon genomics suite version 201808.05 (DNA alignment, sorting, deduplicating, recalibrating, variant calling), SnpSift v4.3, dbSNP build 151, COSMIC v85, CNVkit v0.9.6, BCFtools from Samtools v1.9. PureCN v1.21.21, R v3.5.2, TabNet - open-source PyTorch implementation (https://github.com/dreamquark-ai/tabnet) with PyTorch v1.7.0., Python modules XGBoost v1.2.1 and LightGBM v3.3.2, in Python version 3.8.12.
