# Supplementary material for: Fast, accurate, and racially unbiased pan-cancer tumor-only variant calling with tabular machine learning
Source: NPJ Precis Oncol. 2023 Jan 7;7:4. doi: 10.1038/s41698-022-00340-1 (PMC9825621; doi:10.1038/s41698-022-00340-1)
Supplement: Supplementary file 1 — Supplementary Material [file 41698_2022_340_MOESM1_ESM.pdf]

Supplementary Information for

Fast, accurate, and racially unbiased pan-cancer tumor-only variant calling with tabular machine learning

R. Tyler McLaughlin, Maansi Asthana, Marc Di Meo, Michele Ceccarelli, Howard J. Jacob, David L. Masica

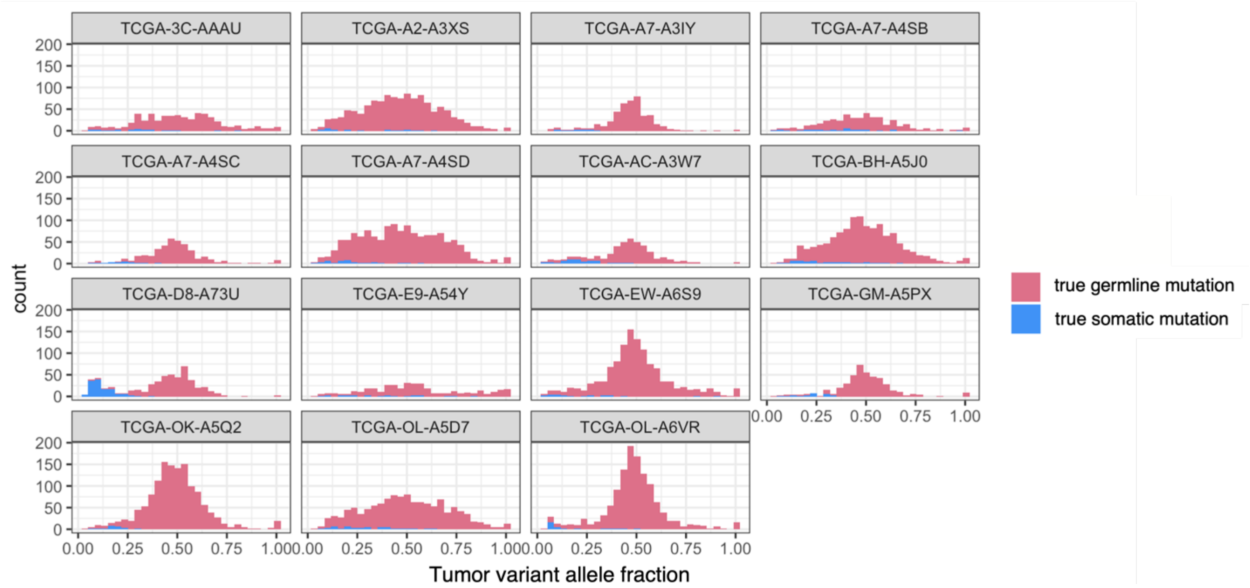

Supplementary Figure 1: The overlapping distributions of variant allele fractions (VAFs) of true somatic and true rare germline variants is a source of complication in tumor-only somatic variant calling. Rare germline variants greatly outnumber true somatic mutations, leading to a high false positive rate in tumor-only somatic variant calling. Data shown are for the 15 breast cancer patients from TCGA included in this study.

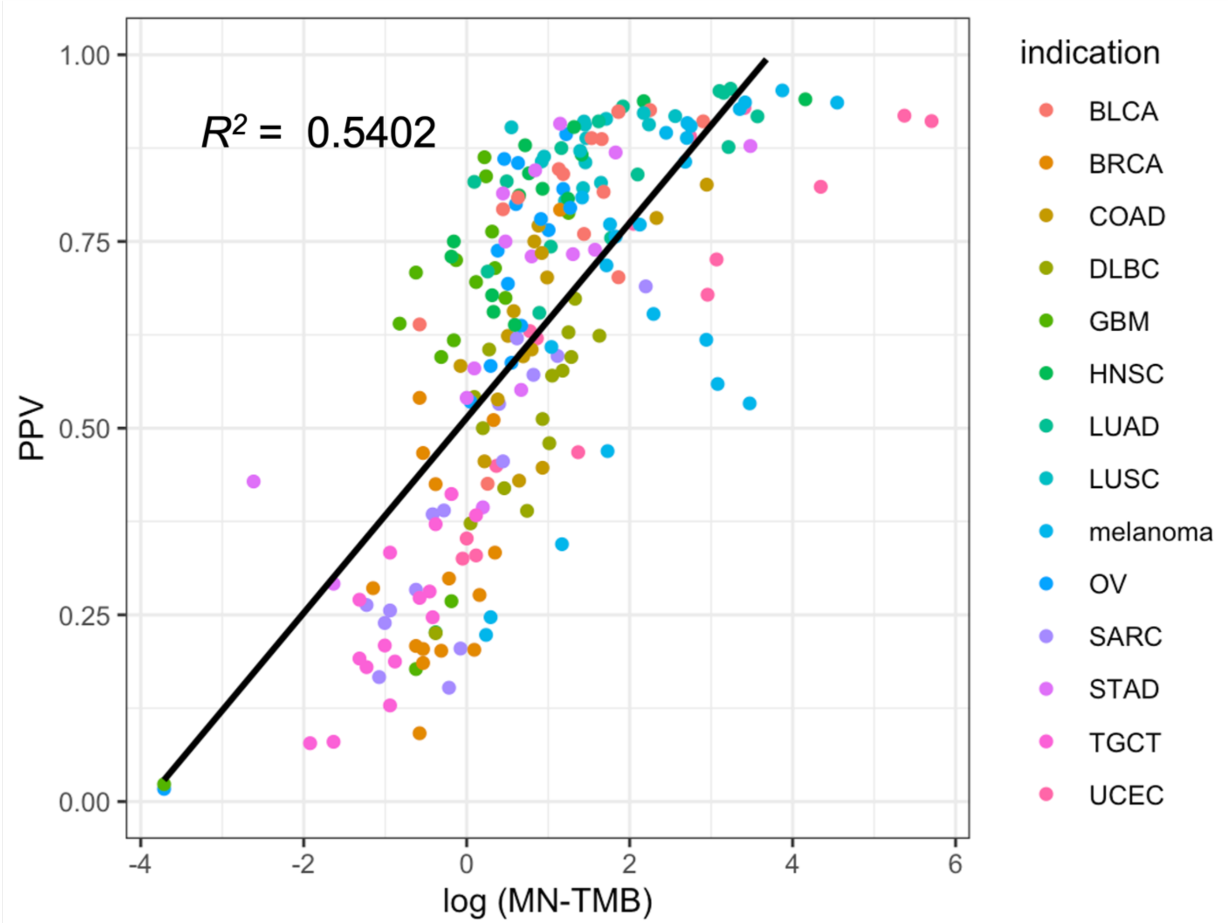

Supplementary Figure 2: Across samples, the positive predictive value (PPV) of the somatic-germline classifier is correlated with the log matched-normal TMB (MN-TMB, the "true" TMB). All 218 samples in this study are displayed.

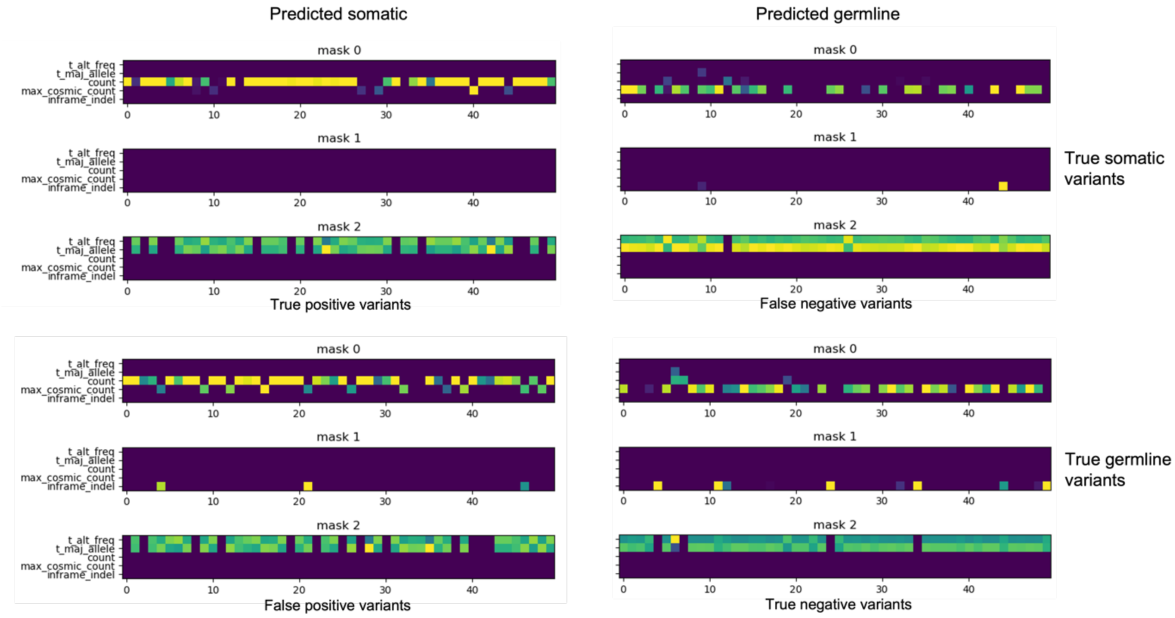

Supplementary Figure 3: TabNet's feature masks show where the neural network allocated its attention during training to arrive at its classification decision. For each of the four variant categories – true positives (*left*), false positives (*top right*), false negatives (*bottom left*), true negatives *(bottom right)* – 50 variants are randomly selected from the TCGA hold out test set. Only the first 3 feature masks are shown, and only the first 5 variables are shown.

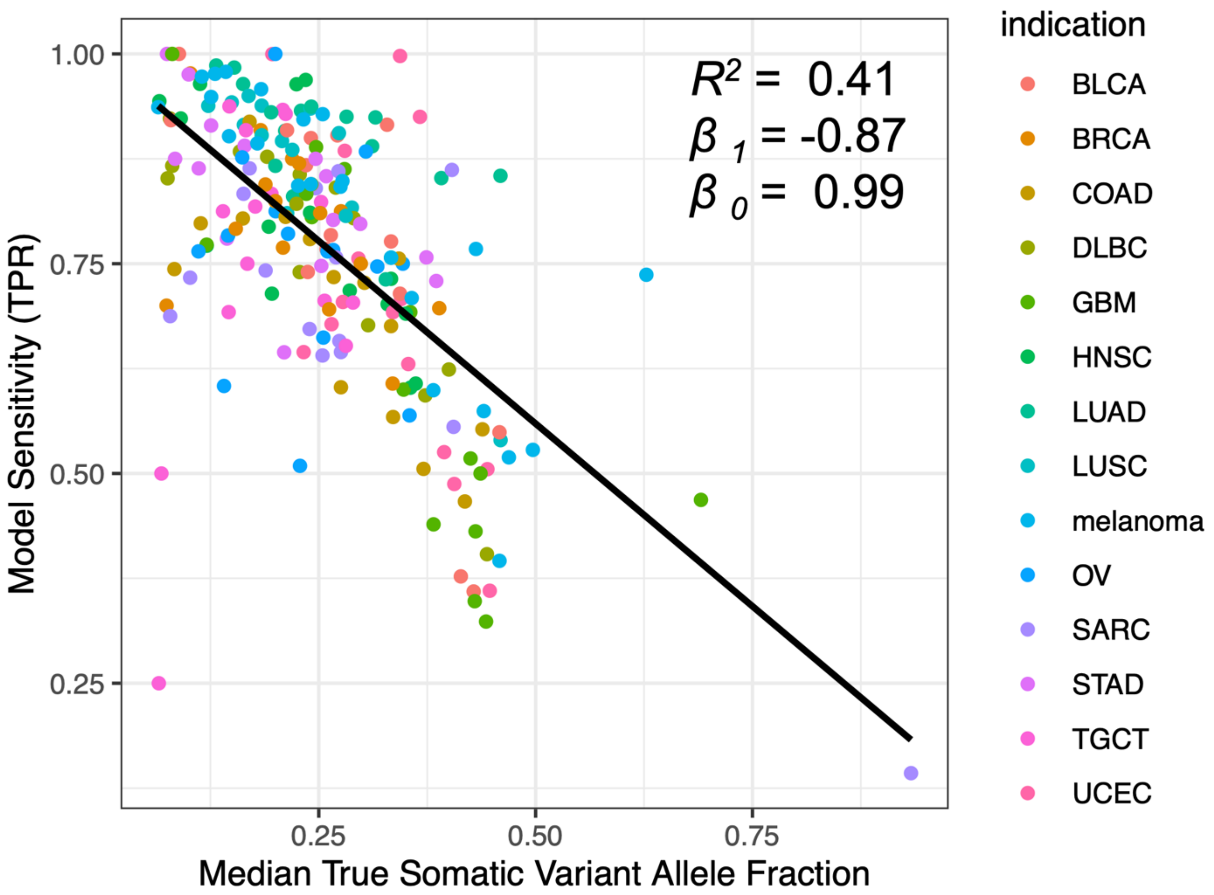

Supplementary Figure 4: Classifier sensitivity (TPR) is negatively correlated with the median variant allele fraction (VAF) of the true somatic mutations (MVTSM), a metric related to tumor purity. All 218 samples in this study are displayed.

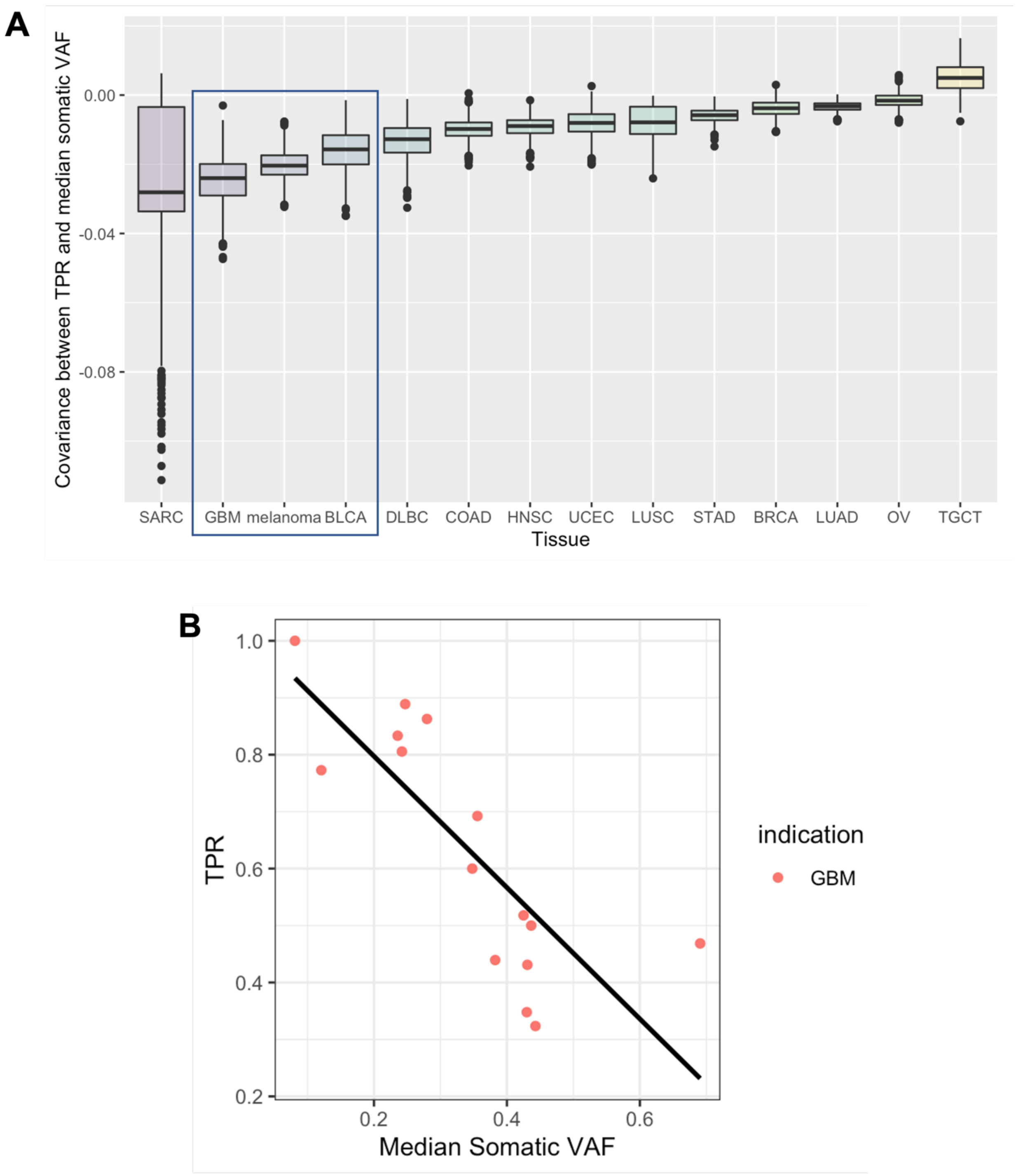

Supplementary Figure 5: A) Bootstrapped covariance analysis ranks the 14 cancer indications in this study by the strength of the inverse relationship between sensitivity (TPR) vs median VAF of true somatic mutations (MVTSM). **B)** Glioblastoma multiforme (GBM) is highlighted as a cancer subtype whose TPR is strongly inversely related to MVTSM.
