# Supplementary material for: Fast, accurate, and racially unbiased pan-cancer tumor-only variant calling with tabular machine learning
Source: NPJ Precis Oncol. 2023 Jan 7;7:4. doi: 10.1038/s41698-022-00340-1 (PMC9825621; doi:10.1038/s41698-022-00340-1)
Supplement: Supplementary file 2 — REPORTING SUMMARY [file 41698_2022_340_MOESM2_ESM.pdf]

nature portfolio

Corresponding author(s): R. Tyler McLaughlin and David L. Masica

Last updated by author(s): Oct 17, 2022

Reporting Summary

Nature Portfolio wishes to improve the reproducibility of the work that we publish. This form provides structure for consistency and transparency in reporting. For further information on Nature Portfolio policies, see our Editorial Policies and the Editorial Policy Checklist.

Statistics

For all statistical analyses, confirm that the following items are present in the figure legend, table legend, main text, or Methods section.

n/a	Confirmed	
☐	☒	The exact sample size (n) for each experimental group/condition, given as a discrete number and unit of measurement
☐	☒	A statement on whether measurements were taken from distinct samples or whether the same sample was measured repeatedly
☐	☒	The statistical test(s) used AND whether they are one- or two-sided<br>*Only common tests should be described solely by name; describe more complex techniques in the Methods section.*
☐	☒	A description of all covariates tested
☐	☒	A description of any assumptions or corrections, such as tests of normality and adjustment for multiple comparisons
☐	☒	A full description of the statistical parameters including central tendency (e.g. means) or other basic estimates (e.g. regression coefficient) AND variation (e.g. standard deviation) or associated estimates of uncertainty (e.g. confidence intervals)
☐	☒	For null hypothesis testing, the test statistic (e.g. F, t, r) with confidence intervals, effect sizes, degrees of freedom and P value noted<br>*Give P values as exact values whenever suitable.*
☒	☐	For Bayesian analysis, information on the choice of priors and Markov chain Monte Carlo settings
☒	☐	For hierarchical and complex designs, identification of the appropriate level for tests and full reporting of outcomes
☐	☒	Estimates of effect sizes (e.g. Cohen's d, Pearson's r), indicating how they were calculated

Our web collection on statistics for biologists contains articles on many of the points above.

Software and code

Policy information about availability of computer code

Data collection	TCGA biolinks version 2.10.5, R version 3.5.2, SRA Toolkit 2.9.6
Data analysis	For DNA processing like alignment and variant calling, we used the Sentieon genomics suite version 201808.05 (alignment, sorting, deduplicating, recalibrating, variant calling), SnpSift v4.3, dbSNP build 151, COSMIC v85, CNVkit v0.9.6, BCFTools from Samtools v1.9. For somatic variant classification, we used PureCN v1.21.21, R v3.5.2, TabNet - open-source PyTorch implementation (https://github.com/dreamquark-ai/tabnet) with PyTorch v1.7.0. Python modules XGBoost v1.2.1 and LightGBM v3.3.2, in Python version 3.8.12.

For manuscripts utilizing custom algorithms or software that are central to the research but not yet described in published literature, software must be made available to editors and reviewers. We strongly encourage code deposition in a community repository (e.g. GitHub). See the Nature Portfolio guidelines for submitting code & software for further information.

Data

Policy information about availability of data

All manuscripts must include a data availability statement. This statement should provide the following information, where applicable:

- Accession codes, unique identifiers, or web links for publicly available datasets
- A description of any restrictions on data availability
- For clinical datasets or third party data, please ensure that the statement adheres to our policy

All genomic and clinical data used in this study are available online. TCGA whole exome sequencing data were acquired through Genomic Data Commons, requiring authorization from dbGaP. Metastatic melanoma whole exome sequencing data were acquired from SRA under the accession number SRP067938.

Human research participants

Policy information about studies involving human research participants and Sex and Gender in Research.

Reporting on sex and gender	N/A
Population characteristics	Relevant patient demographics -- such as race, ethnicity, and cancer subtype -- are provided in Supplemental Table 1. All data used in this study are publicly available, so additional demographics such age can be accessed from TCGA studies and Hugo et al., 2016.
Recruitment	N/A
Ethics oversight	All data used in this study are publicly available. As such, neither patient consent nor institutional review board approval was required.

Note that full information on the approval of the study protocol must also be provided in the manuscript.

Field-specific reporting

Please select the one below that is the best fit for your research. If you are not sure, read the appropriate sections before making your selection.

☒ Life sciences ☐ Behavioural & social sciences ☐ Ecological, evolutionary & environmental sciences

For a reference copy of the document with all sections, see nature.com/documents/nr-reporting-summary-flat.pdf

Life sciences study design

All studies must disclose on these points even when the disclosure is negative.

Sample size	We wanted at least 10 samples for each cancer subtype so we could make conclusions about how biological differences impacted performance of the machine learning classifiers. For our 195 patients from TCGA we had 15 samples for each cancer subtype and across 13 subtypes. For classifying metastatic melanoma mutations, we included 23 patients in the second blind hold-out test set,
Data exclusions	We looked at 13 solid tumor subtypes, including the heme malignancy diffuse large B-cell leukemia We had originally included AML (Acute Myeloid Leukemia) but found the normal samples had somatic mutations (which makes sense in retrospect as this is a liquid tumor type). Thus, we excluded all AML patients from our analysis. We excluded the metastatic patients from Hugo et al., 2016 that were missing capture kit technology information, and so we only included the patients sequenced at UCLA (all 23 of them).
Replication	We used two separate blind hold-out test sets to test that the results are reproducible and generalize to new cancer subtypes. We found performance was even stronger on the Hugo et al. blind holdout test set.
Randomization	Samples were randomly selected given they matched the subtypes of interest and the whole exome sequencing capture kit protocol of interest.
Blinding	Blinding was not relevant, as this is a computational study on genomics data from human participants. We did,however, ensure that our machine learning models were applied to new data outside of the training and validation sets (two "blind hold-out test sets")

Reporting for specific materials, systems and methods

We require information from authors about some types of materials, experimental systems and methods used in many studies. Here, indicate whether each material, system or method listed is relevant to your study. If you are not sure if a list item applies to your research, read the appropriate section before selecting a response.

Materials & experimental systems

n/a	Involved in the study
☒	☐ Antibodies
☒	☐ Eukaryotic cell lines
☒	☐ Palaeontology and archaeology
☒	☐ Animals and other organisms
☒	☐ Clinical data
☒	☐ Dual use research of concern

Methods

n/a	Involved in the study
☒	☐ ChIP-seq
☒	☐ Flow cytometry
☒	☐ MRI-based neuroimaging
